# A preliminary molecular phylogeny of the genus *Scobura*, with a synonym of *Scobura masutaroi* (Lepidoptera, Hesperiidae)

**DOI:** 10.3897/zookeys.638.10026

**Published:** 2016-12-07

**Authors:** Zhen-Fu Huang, Wen Fei, Min Wang, Hideyuki Chiba, Xiao-Ling Fan

**Affiliations:** 1 Department of Entomology, College of Ariculture, South China Agricultural University, Guangzhou 510642, China; 2 B. P. Bishop Museum, 1525 Bernice Street, Honolulu, Hawaii, 96817-0916 U.S.A.

**Keywords:** COI, EF-1α, Scobura masutaroi, Scobura mouchai

## Abstract

A molecular phylogeny of the genus *Scobura* based on the mitochondrial COI and the nuclear EF-1α genes using maximum likelihood and Bayesian inference is proposed. The analyses include 19 specimens from nine ingroup species. The monophyly of *Scobura* is not strongly supported, but two strongly supported monophyletic groups within the genus are recognized: the *Scobura coniata* group and the *Scobura woolletti* group. Judging from combination of the molecular evidence and morphological features, the former consists of six species, including *Scobura masutaroi*, while four species belong to the latter. *Scobura mouchai* Krajcik, 2013 is confirmed to be a **syn. n.** of *Scobura masutaroi* Sugiyama, 1996. The key to the species of the genus *Scobura* is modified to reflect these results.

## Introduction

The skipper genus *Scobura* Elwes & Edwards, 1897 was recently revised by Fan et al. (2010), who recognized 14 species. The genus *Scobura*, however, includes another species, *Scobura masutaroi*, Sugiyama 1996. Fan et al. (2010) overlooked the existence of this taxon and did not include it in their revisional work, which resulted in Krajcik (2013) proposing a new taxon, *Scobura mouchai*, from Shaanxi.

Although a comprehensive morphological revision of the genus has been completed, no phylogenetic analysis has been performed to infer relationships within the genus. In the present study, we present a preliminary phylogeny of *Scobura*, based on molecular evidence. By comparing molecular and morphological evidence, we examine whether *Scobura mouchai* is a synonym of *Scobura masutaroi*.

## Methods

### Morphological examination

See Fan et al. (2010) for materials for the morphological study. In order to examine the wing venation, wings were removed from thorax, cleaned with 95% ethanol, and dyed red with acetocarmine (Wang et al. 2011).

### Taxon sampling

Twenty-three specimens including nine of the 15 valid species of *Scobura* and four outgroup species were included in the phylogenetic reconstruction. Detailed information on the specimens is provided in Table 1. Specimens used in this study were mainly deposited in the Insect Collection, Department of Entomology, South China Agriculture University (SCAU), except for some specimens in Kyushu University museum (KU) and Mr. Hiroaki Onodera’s private collection.

**Table 1.** Voucher information and GenBank accession numbers for the specimens in this study.

Species	Locality	Latitude	Longitude	Voucher Number	COI	EF-1α
*Scobura cephaloides kinka*Evans, 1949	China: Hainan	19.02N	109.53E	SCAU He102	KY049936	KY049958
*Scobura cephaloides kinka*Evans, 1949	Laos: Luang Prabang	19.93N	102.07E	Onodera He553	KY049937	KY049959
*Scobura coniata* Hering, 1918	China: Guangdong	24.91N	113.04E	SCAU He073	KY049938	KY049960
*Scobura coniata* Hering, 1918	China: Guangdong	24.87N	113.03E	SCAU He472	KY049939	KY049961
*Scobura hainana* (Gu & Wang, 1997)	China: Guangdong	24.87N	113.04E	SCAU He471	KY049940	KY049962
*Scobura hainana* (Gu & Wang, 1997)	China: Guangdong	24.87N	113.04E	SCAU He487	KY049941	KY049963
*Scobura hainana* (Gu & Wang, 1997)	China: Guangdong	24.87N	113.04E	SCAU He488	KY049942	KY049964
*Scobura isota* (Swinhoe, 1893)	Thailand: Kanchanaburi	14.08N	99.36E	SCAU He538	KY049943	KY049965
*Scobura isota* (Swinhoe, 1893)	Thailand: Mae Hong Son	19.35N	98.14E	SCAU He468	KY049944	KY049966
*Scobura lyso* (Evans, 1939)	China: Zhejiang	30.15N	119.25E	SCAU He465	KY049945	—
*Scobura lyso* (Evans, 1939)	China: Zhejiang	30.15N	119.25E	SCAU He475	KY049946	—
*Scobura masutaroi* Sugiyama, 1996	China: Sichuan	29.94N	102.48E	SCAU He300	KY049947	KY049967
*Scobura masutaroi* Sugiyama, 1996	China: Sichuan	29.94N	102.48E	SCAU He301	KY049948	KY049968
*Scobura masutaroi* Sugiyama, 1996 (=*mouchai*)	China: Shaanxi	31.91N	106.34E	SCAU He303	KY049949	KY049969
*Scobura parawoolletti* Fan et al., 2010	China: Hainan	19.03N	109.53E	SCAU He116	KY049950	KY049970
*Scobura stellata* Fan et al., 2010	China: Guangdong	24.92N	113.01E	SCAU He036	KY049951	KY049971
*Scobura woolletti* (Riley, 1923)	Indonesia: Kabandungan	6.77 S	106.60E	KU He535	KY049952	KY049972
*Scobura woolletti* (Riley, 1923)	Indonesia: Kabandungan	6.77 S	106.60E	KU He536	KY049953	KY049973
*Scobura woolletti* (Riley, 1923)	Indonesia: Kabandungan	6.77 S	106.60E	KU He537	KY049954	KY049974
*Suastus gremius* (Fabricius, 1798)	China: Guangdong	23.15N	113.34E	SCAU He157	KY049955	KY049975
*Suada swerga* (deNicéville, 1884)	Thailand: Chiang Mai	18.80N	98.92E	SCAU He495	KY049956	KY049976
*Hyarotis quinquepunctatus* Fan & Chiba, 2008	China: Hainan	19.03N	109.54E	SCAU He114	—	KY049977
*Zographetus satwa* (deNicéville, 1884)	China: Guangdong	24.88N	113.03E	SCAU He442	KY049957	KY049978

### Laboratory protocols

Genomic DNA was extracted from the thorax of specimens preserved in ethanol, or from legs of dried specimens, using Magen’s Blood/cell/tissue DNA extraction kit. One mitochondrial gene *cytochrome c oxidase I* (COI) and one nuclear gene *elongation factor 1-α* (EF-1α) were used as molecular phylogenetic markers. The following primers were used for amplification and sequencing in this study: for COI – primers LCO1490 and HCO2198 (Folmer et al. 1994); for EF-1α – primers ef44 and efrcM4 (Monteiro and Pierce 2001). Ploymerase Chain Reaction (PCR) were performed in 20 µl volumes containing 1 µl template DNA, 2 µl 10× buffer, 1.6 µl dNTPs (containing 2.5 mM of each dNTP), 0.8 µl of each primer (10 uM), 0.2 µl Taq Polymerase (2 U/µl), and 13.6 µl ddH_2_O. The PCR Products were amplified using initial denaturation at 94 °C for 4 min, 35 cycles of denaturation at 94 °C for 30 s, annealing at 47 °C (COI) for 45 s, 55 °C (EF-1α) for 1 min, elongation at 72 °C for 1.5 min, and final elongation at 72 °C for 5 min.

Amplified DNA products were purified using an Agarose Gel Extraction kit (Magen Biotech), and directly sequenced, or cloned with pMD18-T vector (Takara Inc), and then sequenced. Sequencing was performed using the ABI 3730 automated sequencer. All sequences were submitted to the Genbank database (accession numbers are given in Table 1).

### Phylogenetic analyses

Alignment of the DNA sequences were performed in Clustal X (Thompson et al. 1997) and edited manually in MEGA 6.0 (Tamura et al. 2013). All base frequencies and molecular character statistics were calculated in MEGA 6.0. Phylogenetic trees were constructed under Maximum Likelihood (ML) and Bayesian inference (BI) criteria. For ML analysis, RAxML version 8 (Stamatakis et al. 2014) was used on a concatenated data set of two genes, with 1000 rapid bootstrap replicates using GTR+G substitution model on the CIPRES Science Gateway (Miller et al. 2010). BI was carried out using Markov Chain Monte Carlo (MCMC) randomization in MrBayes v3.2.3 (Ronquist et al. 2012). We used reversible-jump MCMC to allow for sampling across the entire substitution rate models. Four Markov chains (three heated chains, one cold) were run for 500, 000 generations, with the first 25% of sampled trees discarded as burn-in. The two independent runs were considered to have converged when the standard deviation of split frequencies value was <0.01. The convergence of the analysis was determined in Tracer v1.6 (Rambaut et al. 2014). Bayesian posterior probabilities (PP) and ML
bootstrap values (BP) were used to evaluate branch support.

## Results

### Sequence data

From a total of 23 samples, 22 sequences for COI and 21 for EF-1α were obtained. The alignment of the combined sequences consisted of a total of 1724 bp (658 bp of COI and 1066 bp of EF-1α genes, respectively), including 277 variable and 200 informative sites.

The pairwise P2K distances among the sequences were variable between genes. The ranges of sequence divergences for two loci and ingroup taxa are: COI (0–12.4%), EF-1α (0–5.0%). For COI, sequence divergence between conspecific individuals ranged from 0 to 0.6%; inter-specific genetic distances ranged from 3.6% to 12.4% with divergences among species averaging 7.9% (Table 2).

**Table 2.** Uncorrected pairwise genetic distances (Kimura 2-parameter) for the COI sequences of the genus *Scobura* species.

		1	2	3	4	5	6	7	8	9	10	11	12	13	14	15	16	17	18
1	*Scobura cephaloides* 102																		
2	*Scobura cephaloides* 553	**0.003**																	
3	*Scobura coniata* 73	0.096	0.098																
4	*Scobura coniata* 472	0.096	0.098	**0.000**															
5	*Scobura hainana* 471	0.084	0.085	0.059	0.059														
6	*Scobura hainana* 487	0.084	0.085	0.059	0.059	**0.000**													
7	*Scobura hainana* 488	0.084	0.085	0.061	0.061	**0.002**	**0.002**												
8	*Scobura isota* 468	0.115	0.117	0.122	0.122	0.099	0.099	0.099											
9	*Scobura isota* 538	0.113	0.115	0.118	0.118	0.096	0.096	0.096	**0.006**										
10	*Scobura lyso* 465	0.087	0.089	0.061	0.061	0.039	0.039	0.039	0.105	0.101									
11	*Scobura lyso* 475	0.085	0.087	0.061	0.061	0.036	0.036	0.036	0.101	0.098	**0.003**								
12	*Scobura masutaroi* 300	0.092	0.096	0.059	0.059	0.056	0.056	0.056	0.107	0.103	0.052	0.052							
13	*Scobura masutaroi* 301	0.092	0.096	0.059	0.059	0.056	0.056	0.056	0.107	0.103	0.052	0.052	**0.000**						
14	*Scobura masutaroi* 303	0.092	0.096	0.059	0.059	0.056	0.056	0.056	0.109	0.105	0.052	0.052	**0.002**	**0.002**					
15	*Scobura parawoolletti* 116	0.094	0.097	0.089	0.089	0.087	0.087	0.087	0.112	0.108	0.084	0.084	0.084	0.084	0.084				
16	*Scobura stellata* 36	0.101	0.104	0.104	0.104	0.101	0.101	0.099	0.108	0.105	0.099	0.099	0.092	0.092	0.094	0.070			
17	*Scobura woolletti* 535	0.094	0.097	0.096	0.096	0.085	0.085	0.085	0.124	0.121	0.092	0.092	0.099	0.099	0.099	0.039	0.074		
18	*Scobura woolletti* 536	0.092	0.096	0.094	0.094	0.084	0.084	0.084	0.123	0.119	0.090	0.090	0.097	0.097	0.098	0.038	0.072	**0.002**	
19	*Scobura woolletti* 537	0.094	0.097	0.096	0.096	0.085	0.085	0.085	0.124	0.121	0.092	0.092	0.099	0.099	0.099	0.039	0.074	**0.000**	**0.002**

### Phylogenetic analyses

**Figure 1.**
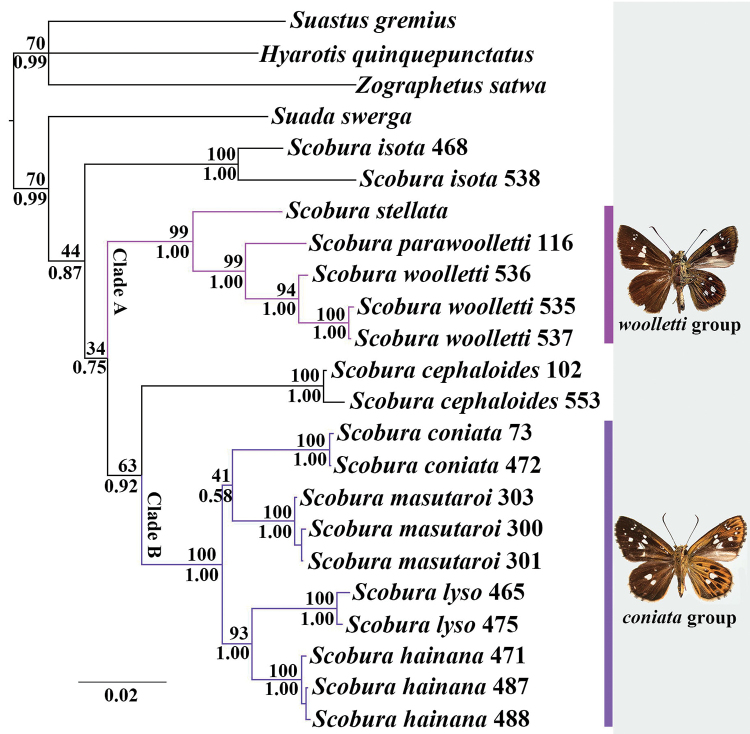
Majority-rule consensus tree from the Bayesian analysis (BI) of the concatenated COI and EF-1α sequences. Values at nodes represent the bootstrap support (BS) values of the maximum likelihood (ML) and the posterior probabilities (PP) of BI analyses, respectively (BP/PP).

The two model-based analyses (BI and ML) revealed nearly identical topologies, differing mainly in branch support (Fig. 1). In both analyses, the monophyly of the genus *Scobura* is weakly supported (BP = 44, PP = 0.87). Within the genus, although support for the basal clades was low, the *Scobura* species included here are clearly distinguished from each other, and formed four clades: the *Scobura isota* clade (which only included two representative specimens), Clade A, the *Scobura cephaloides* clade (only with two representative specimens), and Clade B. Clade A is comprised by *Scobura stellata* + (*Scobura parawoolletti* + *Scobura woolletti*) and receive high bootstrap support and posterior probability (BP = 99, PP = 1.00). We hereafter called the clade *Scobura woolletti* group.

Clade B is comprised by *Scobura masutaroi* and the representatives of *Scobura coniata* group (Devyatkin 2004): *Scobura coniata*, *Scobura lyso* and *Scobura hainana*, and the latter two are sister species with strong support (BP = 93, PP = 1.00). The monophyly of *Scobura coniata* group including *Scobura masutaroi* is strongly supported (BP = 100, PP = 1.00).

In all the analyses, *Scobura cephaloides* is sister to Clade B, with moderate support (BP = 63, PP = 0.92), whereas the relationships between *Scobura isota* and the other clades (Clade A, *Scobura cephaloides* and Clade B) remain unresolved.

## Discussion

**Figure 2.**
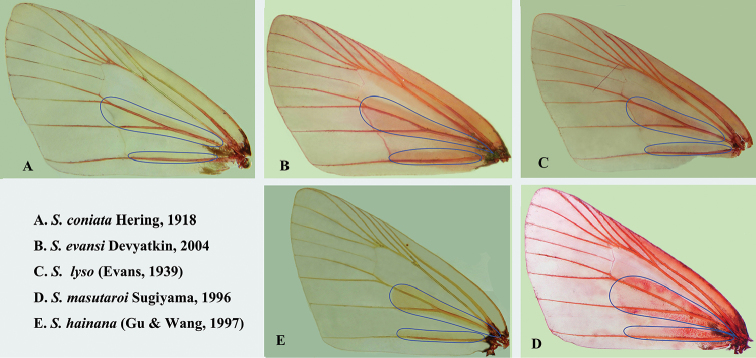
Male band of scent scales in the *Scobura coniata* group species.

Although our phylogenetic analyses do not strongly support the monophyly of the genus *Scobura*, two strongly supported monophyletic groups within the genus are recognized: the *Scobura coniata* group and the *Scobura woolletti* group. The members of the *coniata* group share the following four morphological characters: 1) male band of scent scales on both sides of veins CuA_1_ and CuA_2_ and above 2A on the forewing (Fig. 2); 2) juxta U-shaped with two spine bearing arms, flat at base; 3) tegumen without socius; and 4) uncus thin and long. *Scobura masutaroi* is nested within this group. In our present analyses, two individuals (He 300, 301) of *masutaroi* from Nibashan, Sichuan (close to Dujiangyan, Sichuan, the type locality of *Scobura masutaroi*) and an individual (He303) from Jialingjiang, Fengxian, Shaanxi (the type locality of *Scobura mouchai*) are clearly grouped together with strong support values (BP = 100, PP = 1.00). Moreover, the pairwise P2K distances in COI between the species in the *Scobura coniata* group range from 3.3% to 6.1% with divergences between species averaging 4.5%, while divergence between individuals of *Scobura masutaroi* from Sichuan and Shaanxi province was 0.2%.

Based on the original description, distribution data, and the illustrations provided by Krajcik (2013), as well as our phylogenetic inferences, we conclude that *Scobura mouchai* is identical to *Scobura masutaroi* and should be considered a junior synonym. The male genitalia are illustrated herein, and the female genitalia are described for the first time. On the basis of morphological study (Devyatkin, 2004), two other species, *Scobura phuongi* and *Scobura evani*, which are not included in the present study, likely also belong to this group.

A well-support clade comprised by *Scobura stellata*, *Scobura parawoolletti* and *Scobura woolletti* was recovered in all analyses. These species share the following three characters: 1) hindwing with white spots on underside but not on upperside; 2) socius slender and pointed at tip; and 3) juxta funnel-like, thin and long basally. The generic name *Mimambrix* Riley, 1923 was proposed with *Mimambrix woolletti* as the type species, but later synonymized by Evans (1949). We follow Evans’ treatment and consider this clade as a species group within the genus *Scobura*. Based on morphological characters, the group also includes *Scobura tytleri* (Evans, 1914).

## Taxonomic account

The key given by Fan et al. (2010) is modified to include *Scobura masutaroi*. The couplets leading to *Scobura masuataroi* only are included here. Couplets beyond 11 in the original increase their number by one.

**Table:** 

3	Forewing upper side without spots in spaces M_3_ or M_1_ and M_2_	**4**
–	Forewing upper side with spots in spaces M_1_, M_2_ and M_3_	**6**
4	Forewing upper side without spots in spaces M_1_ and M_3,_ hindwing under side: basal half yellow, distally ferruginous, with five small spots	***Scobura cephaloides***
–	Forewing upper side without spot in space M_3_	**5**
5	Hindwing under side with a conspicuous rectangular white spot in space CuA_2_	***Scobura cephala***
–	Hindwing under side without a conspicuous rectangular white spot in space CuA_2_	***Scobura isota***
6	Hindwing upper side without spot in space CuA_1_, under side with small white spots in spaces Sc+R_1_, M_1-2_, M_3_ and cell	***Scobura eximia***
–	Hindwing upper side with the spot in space CuA_1_	**7**
7	Forewing cell spots conjoined, subequal	**8**
–	Forewing cell spots separated, if conjoined, the lower spot much larger	**9**
8	Hindwing upper side hyaline spots white	***Scobura evansi***
–	Hindwing upper side hyaline spots yellow	***Scobura masutaroi***
9	Forewing upper side the spot in space CuA_2_ triangular, and with a linear stigma crossing the spots in spaces CuA_1_ and CuA_2_	***Scobura coniata***
–	Forewing upper side the spot in space CuA_2_ not as above	**10**
10	Forewing upper side the spot in space CuA_1_ narrow, hindwing upper side without spot in space	***Scobura lyso***
–	Forewing upper side the spot in space CuA_1_ broad	**11**
11	Hindwing upper side spot in space M_3_ tiny dot, forewing upper side cell spots cell spots conjoined	***Scobura hainana***
–	Hindwing upper side spot in space M_3_ significant, forewing upper side cell spots cell spots separated	***Scobura phuongi***

### Scobura masutaroi

Taxon classificationAnimaliaLepidopteraHesperiidae

Sugiyama, 1996

Fig. 3

Scobura masutaroi Sugiyama, 1996: 9 (Type locality: Dujiangyan, Sichuan, China)
Scobura mouchai Krajcik, 2013: 2, **syn. n.** (Type locality: Fengxian, Shaanxi, China)

#### Material examined.

1♂, 1♀, Nibashan, Rongjing, Sichuan, 26.VII.2009, Min Wang; 1♂, Jialingjiang, Fengxian, Shaanxi, 15.VII.2010, Min Wang.

#### Diagnosis.

Forewing length 17–18 mm. This species is different from other species of *Scobura coniata* group in the appearance of the wing upper side: forewing with yellow streak in subcosta space basally, a big cell spots solid across cell, the spot in space CuA_2_ yellow; hindwing with spots in spaces CuA_1_ and M_1_-M_2_ yellow. Wing under side: forewing costal and submarginal spots yellow; hindwing all veins and submarginal spots from spaces Sc+R_1_ to CuA_2_ yellow; and all yellow submarginal spots conjoined both forewing and hindwing.

**Figure 3.**
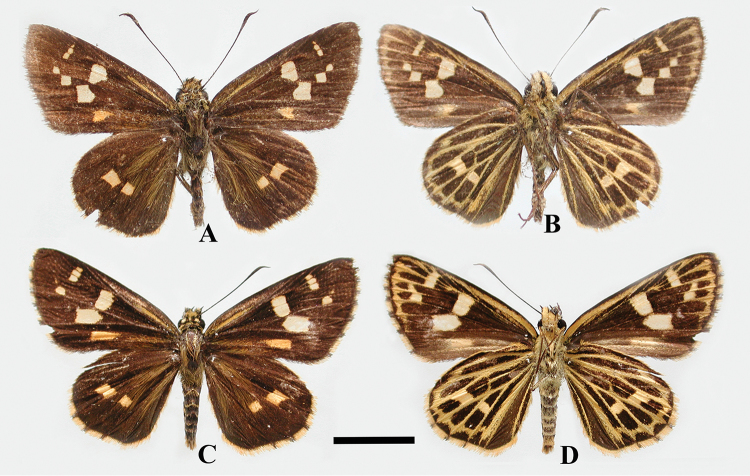
*Scobura masutaroi* Sugiyama, 1996 (Sichuan): **A, B** male **C, D** female; scale bar 10 mm.

#### Description.

**Figure 4.**
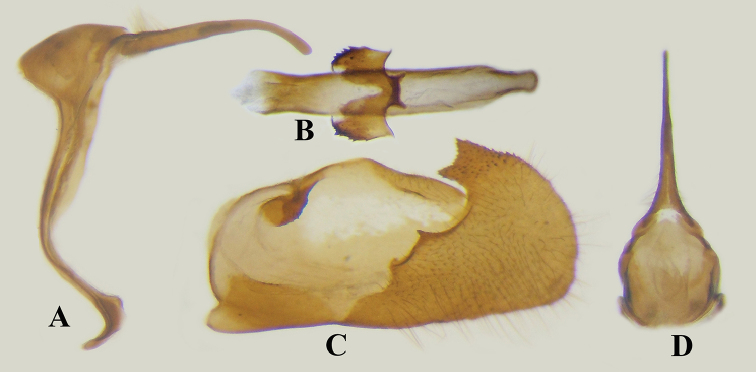
Male genitalia of *Scobura masutaroi* Sugiyama, 1996. (Sichuan). **A** Genitalia ring, lateral view; **B** aedeagus and juxta. **C** valva, inner view; **D** tegument, dorsal view.

Male genitalia (Fig. 4): Tegumen without socius, weakly rounded from lateral view; uncus slender and much longer than tegumen; valva with transtilla rounded and sclerotized with small spines, ventro-distal process irregularly shaped with outer edge rounded, inner edge uneven, and distal part rectangular with densely small spines; saccus short and broad; gnathos absent; juxta U-shaped with two arms with densely spines.

**Figure 5.**
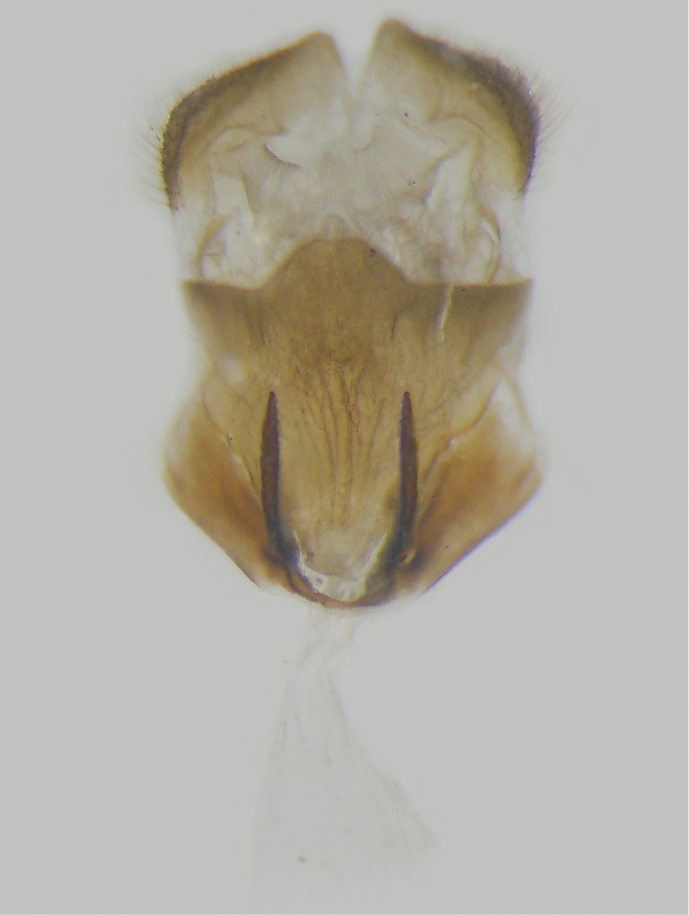
Female genitalia of *Scobura masutaroi* Sugiyama, 1996 (Sichuan)

Female genitalia (Fig. 5): Papillae anales rectangular, covered with setae; anterior lamella U-shaped with sclerotization; posterior lamella triangular with upper margin arched; ductus bursae membranous and short; copulatrix bursa elongate, membranous.

#### Distribution.

China (Sichuan, Shaanxi).

## Supplementary Material

XML Treatment for Scobura masutaroi
